# The use of autogenous tooth bone graft is an efficient method of alveolar ridge preservation – meta-analysis and systematic review

**DOI:** 10.1186/s12903-023-02930-2

**Published:** 2023-04-19

**Authors:** Eleonora Solyom, Eszter Szalai, Márk László Czumbel, Bence Szabo, Szilárd Váncsa, Krisztina Mikulas, Zsombor Radoczy-Drajko, Gabor Varga, Péter Hegyi, Balint Molnar, Reka Fazekas

**Affiliations:** 1 Department of Periodontology, Semmelweis University, Budapest, Hungary; 2 Centre for Translational Medicine, Semmelweis University, Budapest, Hungary; 3 Department of Restorative Dentistry and Endodontics, Semmelweis University, Szentkirályi Utca 47, 1088 Budapest, Hungary; 4 Department of Prosthodontics, Semmelweis University, Budapest, Hungary; 5 Department of Oral Biology, Semmelweis University, Budapest, Hungary; 6 Institute of Pancreatic Diseases, Semmelweis University, Budapest, Hungary; 7 Institute for Translational Medicine, Medical School, University of Pécs, Pécs, Hungary

**Keywords:** Alveolar bone grafting, Alveolar preservation, Alveolar bone loss, Alveolar process, Tooth socket, Socket preservation

## Abstract

**Background:**

Ridge resorption following tooth extraction may be reduced by alveolar ridge preservation (ARP). Previous randomized clinical trials and systematic reviews have suggested that autogenous tooth bone graft (ATB) can be an effective alternative material for ARP. However, the results are heterogeneous. Therefore, our research aimed to evaluate the efficacy of ATB in ARP.

**Methods:**

A systematic search was conducted in Cochrane Library, Embase, MEDLINE and Scopus for studies published from inception to 31 November 2021. We searched searched for randomized, non-randomized controlled trials and case series reporting on ATB use for ARP. The primary outcome was the ridge width difference pre- and post-surgery, measured in millimetres (mm) measured on CBCT (cone beam computed tomography). The secondary outcomes were the histological results. We followed the PRISMA2020 recommendations for reporting our systematic review and meta-analysis.

**Results:**

The analysis included eight studies for the primary and six for the secondary outcomes. The meta-analysis revealed a positive ridge preservation effect with a pooled mean difference ridge width change of -0.72 mm. The pooled mean residual graft proportion was 11.61%, and the newly formed bone proportion was 40.23%. The pooled mean of newly formed bone proportion was higher in the group where ATB originated from both the root and crown of the tooth.

**Conclusions:**

ATB is an effective particulate graft material in ARP. Complete demineralization of the ATB tends to decrease the proportion of newly formed bone. ATB can be an attractive option for ARP.

**Trial registration:**

The study protocol was registered on PROSPERO (CRD42021287890).

**Supplementary Information:**

The online version contains supplementary material available at 10.1186/s12903-023-02930-2.

## Introduction

After tooth extraction a cascade of biological events are triggered that typically result in significant local anatomical changes [1]. Several studies have demonstrated that volume loss after tooth extraction is a natural but irreversible consequence, involving both horizontal and vertical dimension loss, and is most pronounced on the buccal side [2–5]. Without intervention, in the first year the width of the alveolar ridge can be reduced by up to 50% [2, 5, 6].

The hard- and soft-tissue morphology at the extraction site and adjacent teeth determine the course of dental implant placement [5]. Extraction defects are classified according to several grading systems. The severity of the defect is usually categorized by the extent of the buccal bony defect [7, 8], which is the most decisive factor in implant placement. In cases of severe buccal bone loss, alveolar ridge preservation (ARP) might lessen the need for staged surgical rehabilitation. Although alveolar ridge preservation procedures have been used since 1998 there are still debates about its effectiveness [9, 10]. Using ARP, the horizontal and vertical resorption may be reduced by 16–40% [6, 11]. A statistically significant difference can be found between ARP and unassisted healing. However, the clinical significance of it is still unclear [9, 12]. Several techniques and bone grafting materials were advocated for ARP. However, none could fully accomplish the required expectations [13, 14].

For ARP, either particulate or non-particulate graft materials can be utilized. Non-particulate graft materials can complete remodeling but have lower space maintenance properties. The advantage of particulate graft materials is their ease of use and their space-maintaining effect. Using xenografts with a prolonged resorption time has significantly improved alveolar ridge preservation [15]. However, at the time of reentry (at implant placement), graft remnants are frequently detected, potentially interfering with autogenous bone formation and osseointegration of the implant. Some authors reported that none of the graft materials could show higher percentage of newly formed bone proportion than unassisted healing alone [16].

In the 1960s, dentin was evaluated as a biomaterial for inducing bone formation. Bone formation was induced at the tooth extraction sockets and muscles, but only after 8–12 weeks [17]. Since then, numerous preclinical studies have evaluated the biological properties and effects of autogenous tooth bone grafts [18–20]. However, the first human clinical use was only documented in 2010 [21]. The idea was based on the anatomical observation that the embryonic origin of dentin is the same as that of alveolar bone, which may explain its bone-forming capacity [22]. Human dentin and bone are composed of 65% inorganic and 35% organic substances [23]. The inorganic proportion promotes osteoconductivity and space maintenance [24]. On the other hand, the organic matrix of mineralized dentin is responsible for the osteoinductive property [25, 26].

Several protocols have been proposed to produce autogenous tooth bone grafts from extracted teeth, which commonly involve the removal of soft tissues, carious lesions, and fillings after tooth extraction [27]. In addition, some protocols describe the use of only the root of the removed tooth [28], while others recommend using both the crown and the root [29].

According to the degree of demineralization, three main graft types can be distinguished: undemineralized dentin matrix (UDDM), partially demineralized dentin matrix (PDDM), and demineralized dentin matrix (DDM) [30, 31]. Differences between the graft materials and their effect on the healing processes are still under investigation.

Since the first clinical application of ATB, several clinical trials have revealed its potential benefits for ARP. However, clinical studies were conducted with small sample sizes. Therefore, conclusions rely on weak evidence, including high levels of uncertainty. In addition, no meta-analysis has been conducted to confirm the effectiveness of ATB on the preservation of alveolar ridge width; there is also a lack of histological information on its graft remodeling capacity.

This systematic review and meta-analysis aimed to evaluate the current evidence on ATB's efficacy for ARP, the graft turnover capacity, and the effect of utilizing dentin alone vs. dentin combined with enamel to produce ATB.

## Materials and methods

We report our systematic review and meta-analysis based on the recommendation of the PRISMA (Preferred Reporting Items for Systematic reviews and Meta-Analyses) 2020 guideline (see Additional file 1: Appendix Table 1), while we followed the Cochrane Handbook. Furthermore, the study protocol was registered on PROSPERO (International prospective register of systematic reviews; registration number CRD42021287890). We made minor deviations compared to the registered study, however it has no effect on the reported data. The program used for the analysis was changed for easier visualization.

### Eligibility criteria

We used the PICO framework to formulate our research question. We included studies reporting on (P) patients undergoing ARP with (I) particulate ATB graft. The primary outcome (O) was the ridge width change, measured in millimetres (mm). Regarding the change, we radiographically compared (C) the baseline alveolar ridge width to alveolar ridge width 4–6 months postoperatively. Due to the heterogeneity of the measurement methods vertical dimensions of the alveolar ridge could not be investigated. The secondary outcomes were the histological results: the proportion of residual graft, newly formed bone, and connective tissue. We included case series, randomized and non-randomized clinical trials, in which ATB was used in either arm. We excluded literature and systematic reviews and case studies.

Eligible studies included patients over 18 years old with good oral hygiene. ARP was performed with any powder type of autogenous tooth bone graft application with or without membrane coverage, with minimally 3 months of healing. We excluded studies including patients (1) with uncontrolled systemic or infectious diseases, (2) undergoing previous radiotherapy, (3) current or previous bisphosphonate therapy, or (4) heavy smokers (> 5 cigarettes/ day). Studies without linear alveolar ridge width measurement on CBCT or without histomorphometric measurements, immediate implant placement, and those with incomplete data were also excluded.

### Information sources and search strategy

A systematic literature search was conducted in Cochrane Central Register of Controlled Trials (CENTRAL), Embase, MEDLINE (via PubMed), and Scopus for studies published from inception to 31 November 2021. The search key attached to the supplementary material was applied (Additional file 1: Appendix Document 1). The literature search was limited to articles in English.

Furthermore, scanning the bibliographies of all publications selected for our review for inclusion and also the search of the gray literature (expert contact) were accomplished for potentially relevant articles.

### Selection process

Duplicate removal of yielded articles was performed by EndNote X9 (Clarivate Analytics, Philadelphia, PA, USA). Two independent researchers (ES, ESz) followed the Cochrane Handbook’s recommendation and simultaneously screened the titles, abstracts, and full texts of the included studies based on predetermined criteria. The degree of agreement between the review authors was measured using Cohen’s kappa. In case of any disagreement, a consensus was reached after discussion with a third author (BM).

### Data collection process and data items

Data were extracted from the included articles into a pre-defined Excel sheet (Office 365, Microsoft, Redmond, WA, USA) by two authors (ES, ESz) independently. A third party (BM) settled any discrepancies. The following data were collected from each study: first author, article title, study design, demineralization process methods, additional material used (membrane), processing method (root part of tooth vs. whole tooth), measurement method of the preoperative defect morphology.

Primary outcome: mean horizontal ridge width preoperatively and postoperatively, or ridge width changes were measured in mm using CBCT. Secondary outcomes: the proportion of residual graft, newly formed bone and connective tissue in the histological sample expressed in percentage and patient follow-up period.

### Study risk of bias assessment and quality of evidence

Based on the recommendations of the Cochrane Prognosis Methods Group, the ROB-2 (Risk of Bias assessment tool) was used for randomized control trials, and the ROBINS-I (Risk Of Bias In Non-randomized Studies—of Interventions) for non-randomized clinical trials. The methodological quality of the included studies was assessed separately by two authors (ES, ESz). Any disagreement was resolved by arbitration by a third reviewer (BM).

For each analyzed outcome, the certainty of evidence (certainty in the estimates of effect) was determined with the GRADE approach [32].

### Effect measures and statistical analysis

All statistical analyses were made with a preset alpha value of 0.05 using the R (R Core Team 2022; v4.1.1) software and its *meta* (Schwarzer 202; v5.2.0) package. The detailed statistical analysis is presented in the supplementary material (Additional file 1: Appendix Document 2).

We calculated means and mean differences (MDs) with 95% confidence intervals (CIs) from the means and the mean changes of the alveolar crest width and from the histological parameters.

### Subgroup analysis

For the primary outcome a subgroup analysis was conducted according the linear measurement level of the alveolar crest width (subgroup *crest* and *1 mm apically from crest)* (Additional file 1: Appendix Table 3).

For secondary outcomes a subgroup analysis was performed according to the ATB processing methods (Additional file 1: Appendix Table 4). DDM, PDDM and UDDM groups were defined according to the degree of ATB demineralization.

Another subgroup analysis was conducted according to the composition of ATB. The *root* subgroup which originates only from the root portion of the tooth, is composed only of dentin, while the *whole* subgroup which originates from both the root and crown portions of the tooth, is composed of both dentin and enamel (Additional file 1: Appendix Figs. 6–9).

### Certainty of evidence and additional analyses

For each meta-analysis, the certainty of evidence (certainty in the estimates of effect) was determined with the Grading of Recommendations Assessment, Development and Evaluation (GRADE) approach (GRADEpro, 2021) [33]. However, due to the low number of studies, precise outlier and influence analyses could not be carried out. Therefore, we visually inspected funnel plots (Additional file 1: Appendix Figs. 2–5).

## Results

### Search and selection

**Fig. 1:**
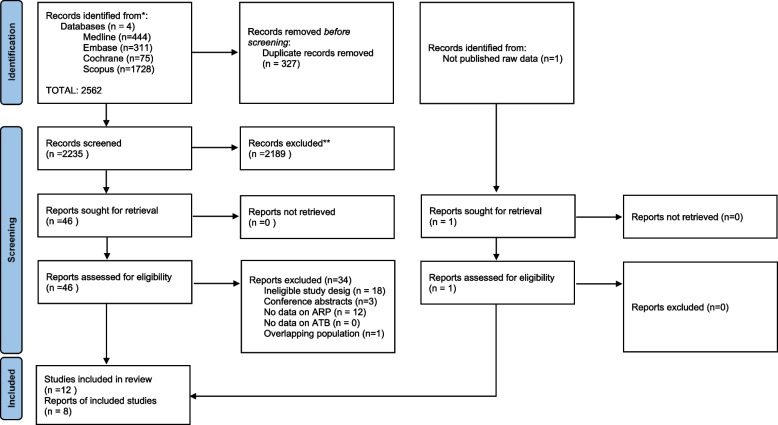
Flowchart of the selection process based on PRISMA 2020 statement. The ‘n’ indicates the total number of studies at each selection level

Our search strategy yielded 2562 studies from the four databases. After duplicate removal, we screened 2235 articles by title and abstract and 46 articles by full text, out of which 12 were eligible for qualitative analysis and eight for meta-analysis. Inter-rater agreement was κ = 0.98. Finally, 12 eligible articles were identified for full-text analysis with an inter-rater agreement of κ = 0.87 (Fig. 1).

### Basic characteristics of included studies

Baseline characteristics of the enrolled analyses are detailed in Table 1. Our quantitative analysis included eight studies with 96 patients for the primary outcome and six studies with 103 patients for the secondary outcome. There was no overlap between the analyzed studies. In four of the six studies, the authors referred to the graft material as a demineralized tooth graft. According to the used demineralization materials and exposure times, two of these can be considered partially demineralized graft material.

**Table 1:** Characteristics of the studies included in the quantitative assessment

Author and year	Study design	Number of patients	Test group	Control group	Follow-up time (months)	Membrane	Used tooth part	Defect morphology	Outcomes
I. W. Um et al. 2019 [34]	case series	T: 10 C: 6	DDM + rhBMP-2	DDM	3–6	no	root	NA	ridge width changes (mm), histological outcomes (%)
C. P. Joshi et al. 2016 [35]	randomized controlled trial	T_1_: 15 T_2_: 15 C: 15	T_1_: ATG T_2_: β-TCP	ungrafted	4	yes	root and crown	min. 5 mm deep, 4 walled bony defects	ridge width changes (mm)
A. Dwivedi et al. 2020 [36]	case series	30	ATG	NA	4	no	root and crown	NA	ridge width changes (mm)
Z. Radoczy-Drajko et al. 2021 [37]	case series	9	ATB	NA	6	yes	root and crown	EDS3-4	ridge width praeop-postop (mm), histological outcomes (%)
A. Elfana et al. 2021 [38]	randomized controlled trial	T: 10 C: 10	AWTG	ADDG	6	yes	root and crown	less than 5 mm buccal bony wall defect	ridge width changes (mm), histological outcomes (%)
G. U. Jung et al. 2018 [39]	randomized controlled trial	T_1_: 8 T_2_: 8 C: 8	T_1_: DDM T_2_: DDM + rhBMP-2	Bio-Oss Collagen	4	yes	root	less than 50% buccal bony defect	ridge width praeop-postop (mm), histological outcomes (%)
K. M. Pang et al. 2017 [40]	randomized controlled trial	T: 21 C: 12	AutoBT	Bio-Oss	6	no	root and crown	min. 4 mm of vertical dimension loss in more than 1 wall	histological outcomes (%)
A. Santos et al. 2021 [29]	randomized controlled trial	T: 34 C: 32	MDM	Bio-Oss	6	yes	root and crown	Elian type II defects	histological outcomes (%)

Of the selected studies, five were RCTs [29, 35, 38–40], and three were clinical trials and case series [34, 36, 37]. Follow-up periods ranged from three to seven months, but in this systematic review and meta-analysis only results between three and six months were recorded.

### Initial defect morphology

Of the selected studies two did not provide any information about the initial defect morphology [34, 36]. Joshi et al. evaluated only four walled bony defects. Drajko et al. included defects with EDS class 3 and 4, which means a minimum 3 mm hard tissue damage at least at one bony wall. Santos et al. used another classification system. They selected extraction defects according to Elian Classification. Elian Type II class means the absence of the middle to the coronal two-third of the labial bone plate of the extraction socket. Elfana et al. evaluated less than 5 mm buccal bony defect, while Pang et al. included defects with a minimum of 4 mm vertical dimensional loss in more than one bony wall. Jung et al. evaluated extraction defects with less than 50% loss of the buccal bony wall.

### Complication, adverse events

In most of the articles no unexpected adverse effects were observed. Jung et al. reported mild pain with the use of DDM in one of the cases. None of the articles reported the need of further augmentation procedures, implant placement was feasible in all reported cases.

Additional details of the study characteristics are found in Additional file 1: Appendix Document 3.

### Risk of bias in studies

The results of the risk of bias assessment are presented in Additional file 1: Appendix Figs. 1–2. The analysis revealed a moderate overall risk of bias in the included studies.

**Fig. 2:**
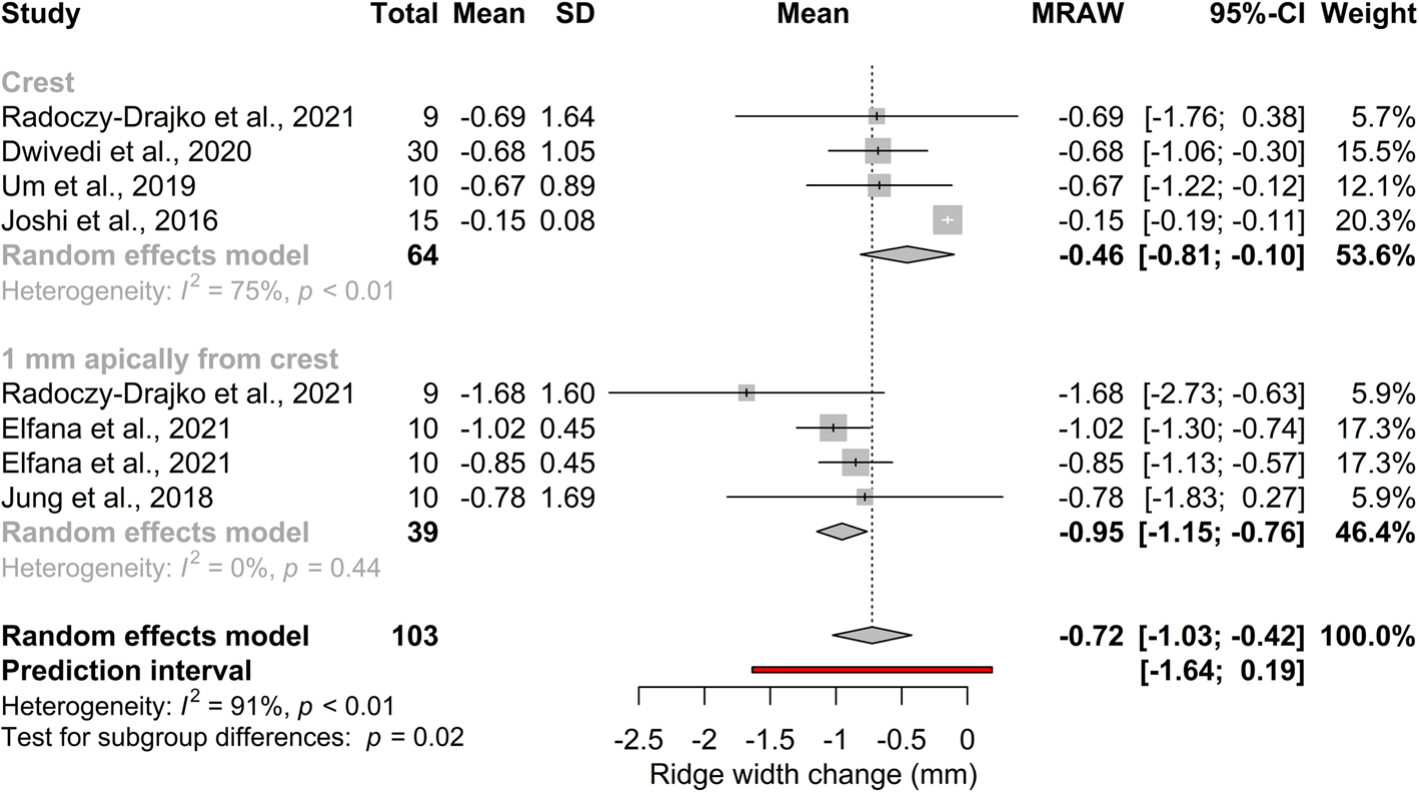
Primary outcome: pooled mean of ridge width changes in mm. A statistically significant alveolar ridge preservation effect can be observed

### Assessment of quality of evidence and additional analyses

The summary of the quality of evidence is included in Additional file 1: Appendix Table 2. In all included studies, horizontal ridge width changes and newly formed and connective tissue proportion generated through GRADEPRO received low certainty values. The reasons for downgrading the quality of evidence were serious imprecision, serious indirectness, and in some cases serious inconsistency. Serious inconsistency occurred due to different measurement methods used across the studies. In the case of indirectness some articles used the whole tooth, and some only the root parts. Small population size was also downgrading factor due to possible imprecision.

The visual inspection of funnel plots (Additional file 1: Appendix Figs. 2–6) indicates the presence of statistical heterogeneity and confounding factors affecting the outcomes.

### Study outcomes

#### Positive horizontal ridge preservation effect

The forest plot in Fig. 2 represents the pooled mean of ridge width changes. Subgroups defined by linear measurement levels are shown in Additional file 1: Appendix Table 3. Quantitative analysis revealed a positive ridge preservation effect at both measurement levels. The overall mean of the ridge width change was -0.72 mm (95% CI[-1.03; -0.42];*I*^*2*^ = 91%; *p*_*I2*_ < 0.01). The effect size was -0.46 mm (95% CI[-0.81; -0.10]; *I*^*2*^ = 75%;*p*_*I2*_ < 0.01;) at the crestal level, and -0.95 mm (95% CI[-1.15; -0.76]; *I*^*2*^ = 0%; *p*_*I2*_ < 0.44) at 1 mm apical from the crestal level. Significant differences were observed between the two subgroups (*p* = 0.02).

#### Promising graft turnover effect

**Fig. 3:**
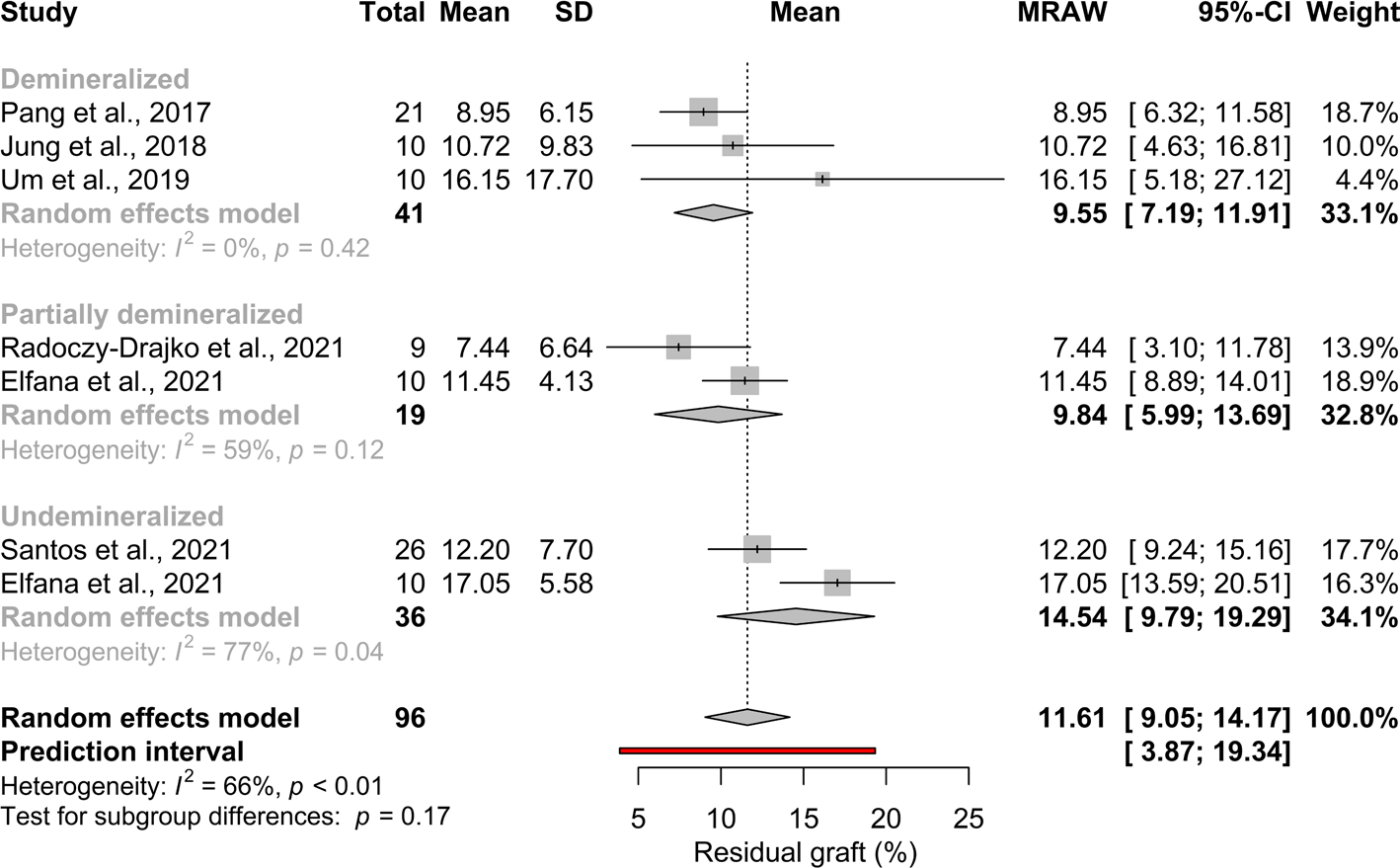
Pooled mean of residual graft proportion (%). No statistical significant difference can be observed between the subgroups

Histological outcomes in the subgroups defined according to the degree of ATB demineralization are shown in Additional file 1: Appendix Table 4. The pooled mean effect size of residual graft proportion was 11.61% (95% CI[ 9.05; 14.17]; *I*^*2*^ = 66%; *p*_*I2*_ < 0.01); (Fig. 3).

#### The degree of ATB demineralization has no effect on graft turnover

No significant difference can be observed between the subgroups: 9.55% (95% CI[ 7.19; 11.91]; *I*^*2*^ = 0%, *p* = 0.42 in DDM; 9.84% (95% CI[ 5.99; 13.69]; *I*^*2*^ = 59%; *p*_*I2*_ = 0.12 in PDDM, and 14.54% (95% CI[ 9.79; 19.29];* I*^*2*^ = 77%; *p*_*I2*_ = 0.04 in UDDM (Fig. 3).

#### The composition of ATB does not affect the graft turnover

The pooled mean of residual graft proportion in the root group and the whole group were 12.00% (95% CI[6.67; 17.33];* I*^*2*^ = 0%; *p*_*I2*_ = 0.40) and 11.27% (95% CI[7.27; 15.26];* I*^*2*^ = 82%; *p*_*I2*_ < 0.01) respectively (Additional file 1: Appendix Fig. 6). No statistically significant difference was observed between the subgroups (*p* = 0.93).

#### Complete demineralization of the graft tends to decrease proportion of the newly formed bone

**Fig. 4:**
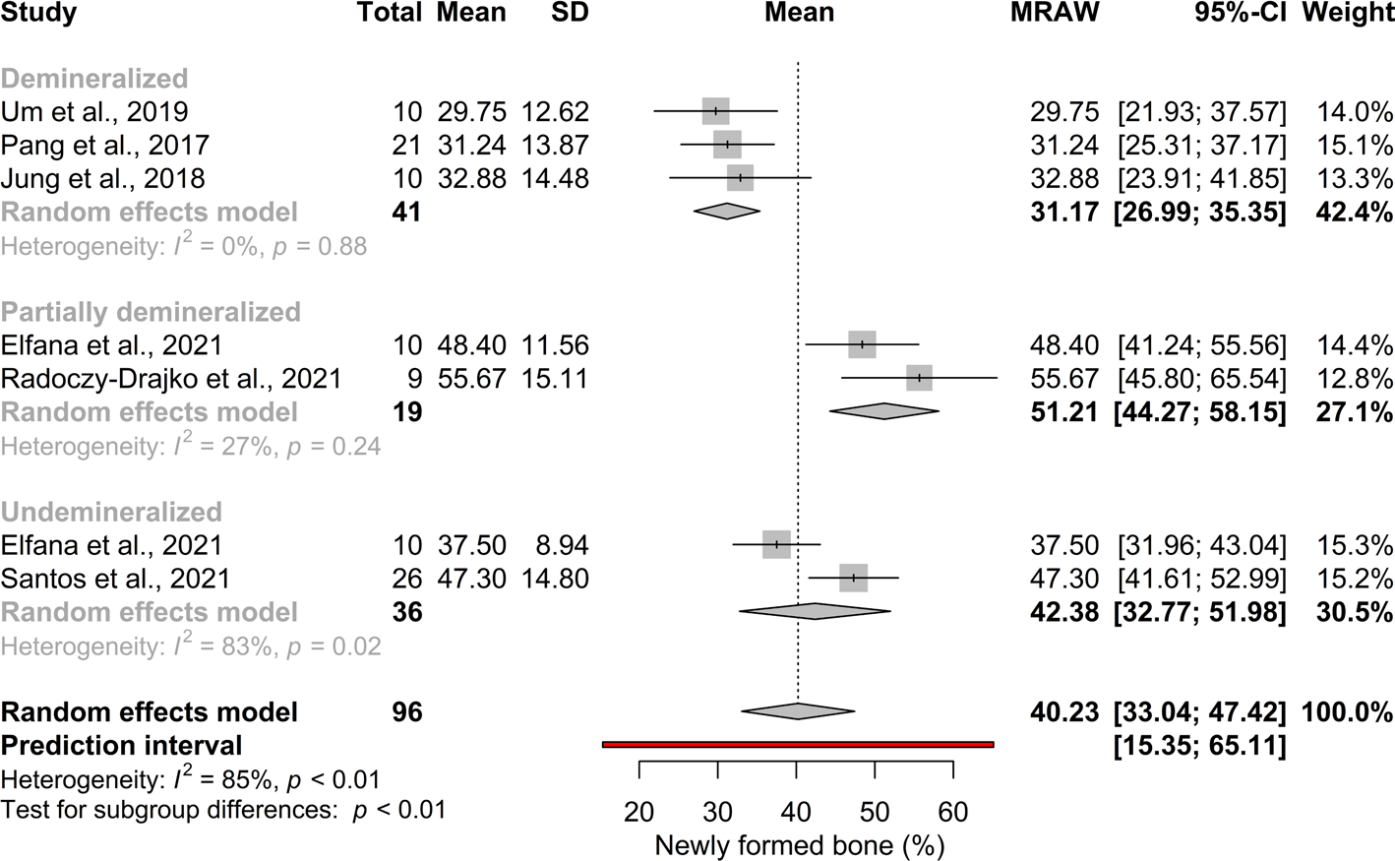
Pooled mean of newly formed bone proportion (%). A statistical significant difference between the DDM and PDDM group can be observed

The pooled mean effect size of newly formed bone proportion was 40.23% (95% CI[ 33.04; 47.42]; *p* < 0.01;), however a considerable heterogeneity was detected (*I*^*2*^ = 85%). The effect size of newly formed bone proportion in the DDM group was 31.17% (95% CI[ 26.99; 35.35]; *I*^*2*^ = 0%; *p*_*I2*_ = 0.88) in the PDDM group was 51.21% (95% CI[ 44.27; 58.15]; *p* = 0.24; *I*^*2*^ = 27%) and in the UDDM group was 42.38% (95% CI[32.77; 51.98]; *p* = 0.02; *I*^*2*^ = 38%) (Fig. 4). There was a statistically significant difference between the DDM and PDDM groups.

#### ATB made from the root and crown parts of the tooth may result a higher amount of newly formed bone

The pooled mean of newly formed bone proportion was higher (*p* < 0.01) in the *whole* group than in the *root* group (42.72% (95% CI[32.24; 53.20];* I*^*2*^ = 88%; *p*_*I2*_ < 0.01 vs. 31.10% (95% CI[25.20; 37.00];* I*^*2*^ = 0%; *p*_*I2*_ = 0.61) (Additional file 1: Appendix Fig. 8).

#### The degree of ATB demineralization may not effect the connective tissue proportion

**Fig. 5:**
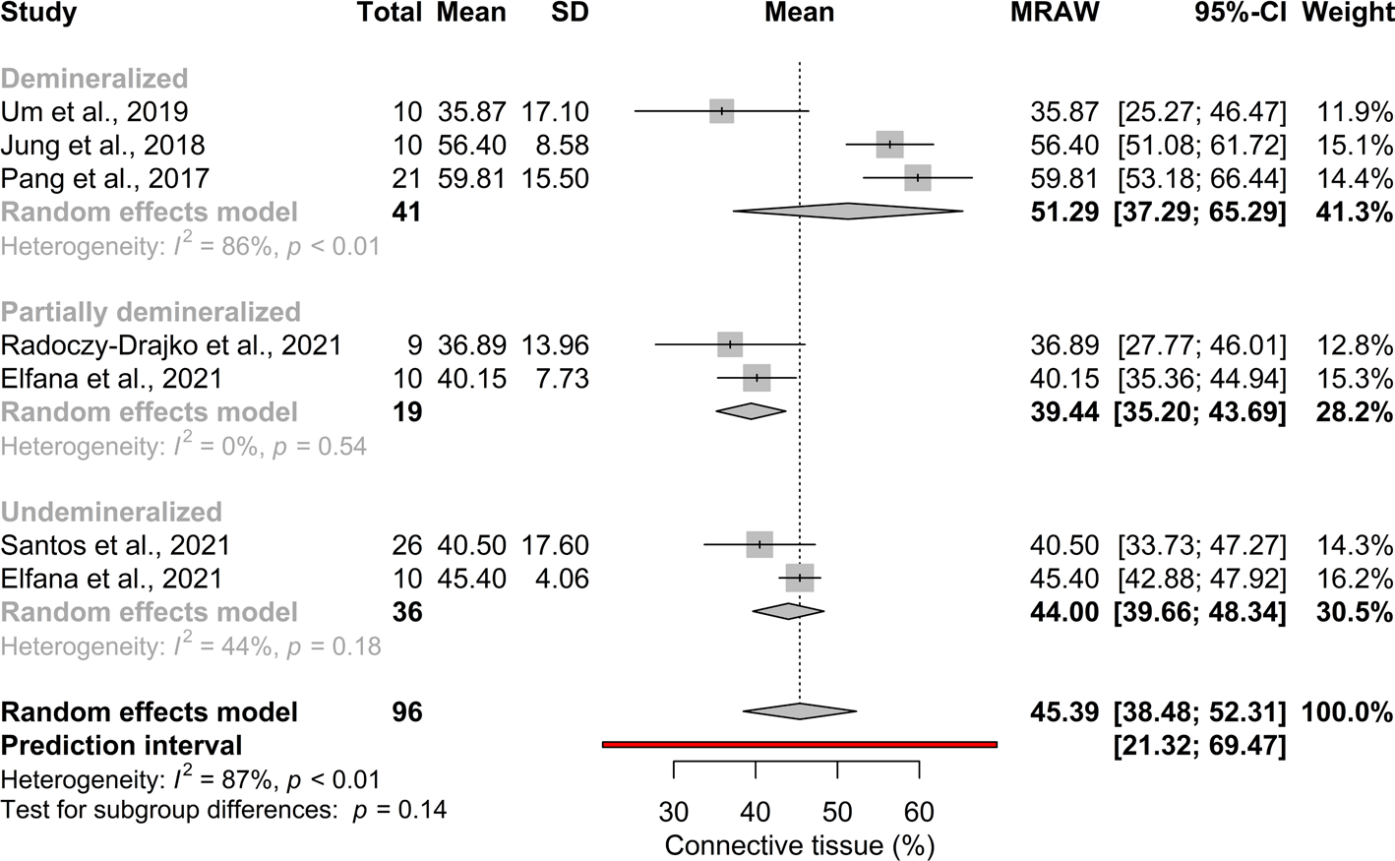
Pooled mean of connective tissue proportion (%). No statistical significant difference can be observed between the subgroups

The pooled mean effect size of connective tissue proportion was 45.39% (95% CI[ 38.48; 52.31]; *p* < 0.01; *I*^*2*^ = 66%) (Fig. 5). A considerable heterogeneity of the subgroups was detected (*I*^*2*^ = 87%). In the DDM group the effect size was 51.29% (95% CI[ 37.29; 65.29]; *I*^*2*^ = 86%; *p*_*I2*_ < 0.01) in the PDDM group 39.44% (95% CI[ 35.20; 43.69]; *I*^*2*^ = 0%; *p*_*I2*_ = 0.54) and 42.38% (95% CI[ 39.66; 48.34]; *I*^*2*^ = 44%; *p*_*I2*_ = 0.18) in the UDDM group. There were no statistically significant differences between subgroups.

#### ATB made from only the root or from the root and crown of the tooth show a similar proportion of connective tissue

In the subgroup analysis based on tooth portions used (Additional file 1: Appendix Fig. 9), the pooled mean effect size in the root group was 46.64% (95% CI[26.58; 66.76];* I*^*2*^ = 91%; *p*_*I2*_ < 0.01) and 45.68% (95% CI[36.10; 55.26];* I*^*2*^ = 89%; *p*_*I2*_ < 0.01) in the root and crown group. No statistically significant difference was observed between subgroups (*p* = 0.63).

## Discussion

Alveolar ridge resorption is an inevitable process, although the extent of tissue breakdown is reducible with appropriate interventions [41, 42]. Several approaches for ARP are available using different graft materials, but none of them deliver ideal outcomes. Since the first use of ARP techniques the popularity of it is still arising. Around 29% of the procedures using grafting materials are ARP surgeries, and it tends to increase approximately 11.4% per year [9]. The cost and also the enviromental footprint of the used graft materials are growing proportionally. Most recently, ATB has been considered an autologous, cost effective, sustainable alternative because it is easily retrievable, safe, and has minimal risk of rejection or infection [43].

All recent systematic reviews on the topic [27, 30, 44, 45] summarized that ATB has a beneficial effect on alveolar ridge preservation, but to the best of our knowledge none of them could statistically demonstrate these findings. Our meta-analysis aimed to statistically confirm this observation regarding changes in alveolar ridge width and histological outcome. Unfortunately, vertical dimensional changes could not be analyzed in this study due to the large differences in measurement methods.

The differences in initial defect morphology, surgical techniques, preparation procedures of ATB, and follow-up time made comparisons of primary studies difficult. Nevertheless, some important findings were made.

In a recent meta-analysis, the change in alveolar ridge width using the xenograft Bio-Oss® material was -0.88 mm [16]. Our data provided similar results (-0.72 mm), suggesting that ATB is as effective in preserving alveolar ridge width as the most studied particulate graft material, although RCTs are needed to directly compare the two materials statistically. An increased heterogeneity is observed, caused by different measurement methods and patient populations with different initial defect morphologies. For example Joshi et al. included extraction defects with four walls, which might have better healing potential than extraction defects with fewer bony walls.

Due to the heterogeneity of the measurement techniques, the present MA cannot compare the reduction in socket dimensional changes following ARP using ATB with extraction alone. However, Del Canto-Díaz et al. found a significant ridge preservation effect in their split-mouth study, with a mean bone loss of vestibular width of 0.46 mm in the ARP group using ATB compared with 1.91 mm in the unprovided extraction sockets group measured from the vertical line to the buccal cortical bone at 1 mm apical from the alveolar crest.

Other hot issues are the rate of graft material resorption and whether intra-socket grafts compromise the normal healing process of the tooth extraction socket. Regarding the resorption rate of ATB graft material, it can be said that ATB may also has greater graft remodelling capacity compared to other particulate graft materials. De Risi et al. in a meta-analysis compared different grafting materials for ARP and concluded that the newly formed bone proportion of xenografts was 23%, the residual graft proportion was 37%, and the connective tissue proportion was 32% [46]. According to our findings, the mean newly formed bone proportion of ATB was 40%, the residual graft proportion was 12% and the connective tissue proportion was 45%.

Due to the low number of studies, a conclusion regarding the efficacy of the processing methods resulting in different levels of ATB mineralization (UDDM, PDDDM, DDM) cannot be drawn, but a slight difference between the outcomes can be observed.

Our meta-analysis shows that the newly formed bone proportion was highest in the PDDM group (51%) and lowest in the DDM group (31%) –a statistically and clinically significant difference. We also found that the proportion of connective tissue was the lowest in the PDDM group (39%) and highest in the DDM group (51%). However, this difference was not significant. These data suggest that partial demineralization may positively affect the rate of new bone formation. This is likely due to increased osteoinductivity resulting from the more exposed collagenous and non-collagenous proteins and growth factors, and increased osteoconductivity resulting from the increased porosity and surface area [43]. However, aggressive demineralization can cause a depletion of growth factors and lead to collapse of the 3D structure [47]. This correlates well with previously conducted preclinical [47] and clinical studies [21, 48–50].

Mazor et al. used UDDM for ARP in combination with a non-resorbable membrane. After seven months, 63% of newly formed bone could be detected. Minetti et al. used DDM for ARP and a xenogenic resorbable membrane for the graft coverage. After four months the total bone volume was 41%. The heterogeneity of measurement techniques means that the current MA cannot use these data either. However, considering the differences in the study designs and measurement parameters, this data suggests that the demineralization of the graft material tends to decrease the newly formed bone proportion [28, 51] and this is in line with our findings.

No statistically significant difference was observed between the subgroups in terms of residual graft remnants. However, the difference in the residual graft proportion between DDM (9.5%), PDDM (9.8%) vs. UDDM groups (14.5%) may be clinically relevant.

According to our analysis, the origin and composition of ATB, i.e., root composed by denin only or root and crown, composed by dentin and enamel, can affect the quantity of newly formed bone. We found a statistically significant difference between the subgroups in favor of the whole tooth group. There was no statistical difference between the subgroups in the graft turnover or connective tissue proportions. The combined application of root and crown also resulted in significantly higher graft volume, increasing the cost/benefit ratio of the treatment.

### Strengths and limitations

Previous systematic reviews collected the available literature data but have not been able to analyze the effects of ATB in alveolar ridge preservation [27, 30, 44, 45, 52]. Regarding the strengths of our analysis, we followed a strict protocol, which was registered in advance. A rigorous methodology was applied. Moreover, the full spectrum of currently available data was analyzed.

However, the results should be interpreted with caution due to the following limitations. The heterogeneity of preparation methods resulting in different ATB composition and mineralization, the difference of initial defect morphology and follow-up times between studies prevents a complete overview of the entire healing process. The presence of a moderate risk of bias in some of the domains is another limitation.

### Clinical and research implications

Based on our results, we suggest more detailed inclusion criteria, randomization procedures, standardized dentin matrix processing methods, and a pre-specified analysis plan. Due to its cost effectiveness and sustainability ATB can be an alternative graft material for ARP.

## Conclusion

Based on our findings, the ATB graft can be an alternative cost-effective and sustainable biomaterial for alveolar ridge preservation. However, further studies with longer follow-up times are needed to evaluate the graft material's long-term stability.

## Supplementary Information

**Additional file 1:** **Appendix Table 1.** PRISMA 2020 Main Checklist. **Appendix Table 2.** Certainty of evidence for each meta-analysis based on the GRADE approach. **Appendix Table 3.** Changes in alveolar ridge width (mean ± standard deviation [SD]). Raw data of Radoczy Drajko et al. was also used (measurement at the crestal level). **Appendix Table 4.** Histological outcomes (mean ± standard deviation [SD]). **Appendix Figure 1.** Quality assessment based on Cochrane Risk of Bias Tool 2 (RoB 2) for randomized controlled trials. **Appendix Figure 2.** Quality assessment based on Cochrane risk of bias tool to assess non-randomized studies of interventions (ROBINS-I) for non-randomized studies of interventions. **Appendix Figure 3.** Funnel plots indicates the presence of statistical heterogeneity and cofounding factors affecting the primary outcome (alveolar ridge width changes). **Appendix Figure 4.** Funnel plots indicates the presence of statistical heterogeneity and cofounding factors affecting the residual graft proportion (%). **Appendix Figure 5.** Funnel plots indicates the presence of statistical heterogeneity and cofounding factors affecting the newly formed bone proportion (%). **Appendix Figure 6.** Funnel plots indicates the presence of statistical heterogeneity and cofounding factors affecting the connective tissue proportion (%). **Appendix Figure 7.** Pooled mean of residual graft proportion. Neither clinical, nor statistical significant difference can be observed between the subgroups. **Appendix Figure 8.** Pooled mean of newly formed bone proportion. A statistical significant difference can be observed between the subgroups. **Appendix Figure 9.** Pooled mean of connective tissue proportion. No statistical significant difference can be observed between the subgroups. **Appendix Document 1.** Search key. **Appendix Document 2.** Detailed statistical analysis. **Appendix document 3.** Detailed study charachteristics.

## Data Availability

The datasets used in this study can be found in the published full-text articles included in the systematic review and meta-analysis.

## References

[1] Van der Weijden F, Dell'Acqua F, Slot DE (2009). Alveolar bone dimensional changes of post-extraction sockets in humans: a systematic review. J Clin Periodontol.

[2] Araujo M, Linder E, Lindhe J (2009). Effect of a xenograft on early bone formation in extraction sockets: an experimental study in dog. Clin Oral Implants Res.

[3] Araujo MG, Lindhe J (2005). Dimensional ridge alterations following tooth extraction. An experimental study in the dog. J Clin Periodontol.

[4] Pietrokovski J, Sorin S, Hirschfeld Z (1976). The residual ridge in partially edentulous patients. J Prosthet Dent.

[5] Schropp L, Wenzel A, Kostopoulos L, Karring T (2003). Bone healing and soft tissue contour changes following single-tooth extraction: a clinical and radiographic 12-month prospective study. Int J Periodontics Restorative Dent.

[6] Lekovic V, Camargo PM, Weinlaender M, Vasilic N, Aleksic Z, Kenney EB (2003). Effectiveness of a combination of platelet-rich plasma, bovine porous bone mineral and guided tissue regeneration in the treatment of mandibular grade II molar furcations in humans. J Clin Periodontol.

[7] Caplanis N, Lozada JL, Kan JY (2005). Extraction defect assessment, classification, and management. J Calif Dent Assoc.

[8] Elian N, Cho SC, Froum S, Smith RB, Tarnow DP (2007). A simplified socket classification and repair technique. Pract Proced Aesthet Dent.

[9] Adams RJ (2022). Is there clinical evidence to support alveolar ridge preservation over extraction alone? A review of recent literature and case reports of late graft failure. Br Dent J.

[10] Atieh MA, Alsabeeha NHM, Payne AGT, Duncan W, Faggion CM, Esposito M (2015). Interventions for replacing missing teeth: alveolar ridge preservation techniques for dental implant site development. Cochrane Database of Systematic Reviews.

[11] Iasella JM, Greenwell H, Miller RL, Hill M, Drisko C, Bohra AA, Scheetz JP (2003). Ridge preservation with freeze-dried bone allograft and a collagen membrane compared to extraction alone for implant site development: a clinical and histologic study in humans. J Periodontol.

[12] MacBeth N, Trullenque-Eriksson A, Donos N, Mardas N (2017). Hard and soft tissue changes following alveolar ridge preservation: a systematic review. Clin Oral Implant Res.

[13] Nampo T, Watahiki J, Enomoto A, Taguchi T, Ono M, Nakano H, Yamamoto G, Irie T, Tachikawa T, Maki K (2010). A new method for alveolar bone repair using extracted teeth for the graft material. J Periodontol.

[14] Nasr HF, Aichelmann-Reidy ME, Yukna RA (2000). Bone and bone substitutes. Periodontol.

[15] Araujo MG, Linder E, Lindhe J (2011). Bio-Oss collagen in the buccal gap at immediate implants: a 6-month study in the dog. Clin Oral Implants Res.

[16] Canellas J, Soares BN, Ritto FG, Vettore MV, Vidigal Júnior GM, Fischer RG, Medeiros PJD (2021). What grafting materials produce greater alveolar ridge preservation after tooth extraction? A systematic review and network meta-analysis. J Craniomaxillofac Surg.

[17] Yeomans JD, Urist MR (1967). Bone induction by decalcified dentine implanted into oral, osseous and muscle tissues. Arch Oral Biol.

[18] Catanzaro-Guimaraes SA, Catanzaro Guimaraes BP, Garcia RB, Alle N (1986). Osteogenic potential of autogenic demineralized dentin implanted in bony defects in dogs. Int J Oral Maxillofac Surg.

[19] Gomes MF, dos Anjos MJ, Nogueira TO, Guimaraes SA (2001). Histologic evaluation of the osteoinductive property of autogenous demineralized dentin matrix on surgical bone defects in rabbit skulls using human amniotic membrane for guided bone regeneration. Int J Oral Maxillofac Implants.

[20] Kim YK, Kim SG, Oh JS, Jin SC, Son JS, Kim SY, Lim SY (2011). Analysis of the inorganic component of autogenous tooth bone graft material. J Nanosci Nanotechnol.

[21] Kim YK, Kim SG, Byeon JH, Lee HJ, Um IU, Lim SC, Kim SY (2010). Development of a novel bone grafting material using autogenous teeth. Oral Surg Oral Med Oral Pathol Oral Radiol Endod.

[22] Melek LN, El Said MM (2017). Evaluation of “Autogenous Bioengineered Injectable PRF – Tooth graft” combination (ABIT) in reconstruction of maxillary alveolar ridge defects: CBCT volumetric analysis. Saudi J Dent Res.

[23] Kim TW, Seo EW, Song SI (2013). Open reduction and internal fixation of mandibular fracture in an 11-month-old infant: a case report. J Korean Assoc Oral Maxillofac Surg.

[24] Wood RA, Mealey BL (2012). Histologic comparison of healing after tooth extraction with ridge preservation using mineralized versus demineralized freeze-dried bone allograft. J Periodontol.

[25] Kim YK, Pang KM, Yun PY, Leem DH, Um IW (2017). Long-term follow-up of autogenous tooth bone graft blocks with dental implants. Clin Case Rep.

[26] Wennerberg A, Albrektsson T, Lindhe J (2003). Clinical periodontology and implant dentistry. Surface Topography of Titanium Dental Implants.

[27] Cenicante J, Botelho J, Machado V, Mendes JJ, Mascarenhas P, Alcoforado G, Santos A (2021). The use of autogenous teeth for alveolar ridge preservation: a literature review. Appl Sci.

[28] Minetti E, Taschieri S, Corbella S (2020). Autologous deciduous tooth-derived material for alveolar ridge preservation: a clinical and histological case report. Case Rep Dent.

[29] Santos A, Botelho J, Machado V, Borrecho G, Proença L, Mendes JJ, Mascarenhas P, Alcoforado G (2021). Autogenous mineralized dentin versus xenograft granules in ridge preservation for delayed implantation in post-extraction sites: a randomized controlled clinical trial with an 18 months follow-up. Clin Oral Implants Res.

[30] Gual-Vaqués P, Polis-Yanes C, Estrugo-Devesa A, Ayuso-Montero R, Mari-Roig A, López-López J (2018). Autogenous teeth used for bone grafting: a systematic review. Med Oral Patol Oral Cir Bucal.

[31] Tabatabaei FS, Tatari S, Samadi R, Moharamzadeh K (2016). Different methods of dentin processing for application in bone tissue engineering: a systematic review. J Biomed Mater Res A.

[32] Kirmayr M, Quilodrán C, Valente B, Loezar C, Garegnani L, Franco JVA (2021). The GRADE approach, part 1: how to assess the certainty of the evidence. Medwave.

[33] Higgins JPT, Thomas J, Chandler J, Cumpston M, Li T, Page MJ, Welch VA (editors). Cochrane Handbook for Systematic Reviews of Interventions version 6.3 (updated February 2022). Cochrane, 2022. Available from www.training.cochrane.org/handbook.

[34] Um I-W, Kim Y-K, Park J-C, Lee J-H (2019). Clinical application of autogenous demineralized dentin matrix loaded with recombinant human bone morphogenetic-2 for socket preservation: A case series. Clin Implant Dent Relat Res.

[35] Joshi CP, Dani NH, Khedkar SU (2016). Alveolar ridge preservation using autogenous tooth graft versus beta-tricalcium phosphate alloplast: a randomized, controlled, prospective, clinical pilot study. J Indian Soc Periodontol.

[36] Dwivedi A, Kour M (2020). A neoteric procedure for alveolar ridge preservation using autogenous fresh mineralized tooth graft prepared at chair side. J Oral Biol Craniofac Res.

[37] Radoczy-Drajko Z, Windisch P, Svidro E, Tajti P, Molnar B, Gerber G (2021). Clinical, radiographical and histological evaluation of alveolar ridge preservation with an autogenous tooth derived particulate graft in EDS class 3–4 defects. BMC Oral Health.

[38] Elfana A, El-Kholy S, Saleh HA, Fawzy El-Sayed K (2021). Alveolar ridge preservation using autogenous whole-tooth versus demineralized dentin grafts: a randomized controlled clinical trial. Clin Oral Implants Res.

[39] Jung GU, Jeon TH, Kang MH, Um IW, Song IS, Ryu JJ, Jun SH. Volumetric, radiographic, and histologic analyses of demineralized dentin matrix combined with recombinant human bone morphogenetic protein-2 for ridge preservation: a prospective randomized controlled trial in comparison with xenograft. Appl Sci. 2018;8(8).

[40] Pang KM, Um IW, Kim YK, Woo JM, Kim SM, Lee JH (2017). Autogenous demineralized dentin matrix from extracted tooth for the augmentation of alveolar bone defect: a prospective randomized clinical trial in comparison with anorganic bovine bone. Clin Oral Implants Res.

[41] Atieh MA, Alsabeeha NH, Payne AG, Ali S, Faggion CMJ, Esposito M (2021). Interventions for replacing missing teeth: alveolar ridge preservation techniques for dental implant site development. Cochrane Database Syst Rev.

[42] Avila-Ortiz G, Elangovan S, Kramer KW, Blanchette D, Dawson DV (2014). Effect of alveolar ridge preservation after tooth extraction: a systematic review and meta-analysis. J Dent Res.

[43] Qin X, Raj RM, Liao XF, Shi W, Ma B, Gong SQ, Chen WM, Zhou B (2014). Using rigidly fixed autogenous tooth graft to repair bone defect: an animal model. Dent Traumatol.

[44] Mahardawi B, Rochanavibhata S, Jiaranuchart S, Arunjaroensuk S, Mattheos N, Pimkhaokham A (2023). Autogenous tooth bone graft material prepared chairside and its clinical applications: a systematic review. Int J Oral Maxillofac Surg.

[45] Ramanauskaite A, Obreja K, Sader R, Khoury F, Romanos G, Koo KT, Keeve PL, Sculean A, Schwarz F (2019). Surgical treatment of periimplantitis with augmentative techniques. Implant Dent.

[46] De Risi V, Clementini M, Vittorini G, Mannocci A, De Sanctis M (2015). Alveolar ridge preservation techniques: a systematic review and meta-analysis of histological and histomorphometrical data. Clin Oral Implants Res.

[47] Koga T, Minamizato T, Kawai Y, Miura K, I T, Nakatani Y, Sumita Y, Asahina I (2016). Bone regeneration using dentin matrix depends on the degree of demineralization and particle size. PLoS One.

[48] Jeong HR, Hwang JH, Lee JK (2011). Effectiveness of autogenous tooth bone used as a graft material for regeneration of bone in miniature pig. jkaoms.

[49] Kim YK, Kim SG, Yun PY, Yeo IS, Jin SC, Oh JS, Kim HJ, Yu SK, Lee SY, Kim JS (2014). Autogenous teeth used for bone grafting: a comparison with traditional grafting materials. Oral Surg Oral Med Oral Pathol Oral Radiol.

[50] Lee JY, Kim YK, Yi YJ, Choi JH (2013). Clinical evaluation of ridge augmentation using autogenous tooth bone graft material: case series study. J Korean Assoc Oral Maxillofac Surg.

[51] Minetti E, Palermo A, Ferrante F, Schmitz JH, Lung Ho HK, Dih Hann SN, Giacometti E, Gambardella U, Contessi M, Celko M (2019). Autologous tooth graft after endodontical treated used for socket preservation: a multicenter clinical study. Appl Sci.

[52] Del Canto-Díaz A, de Elío-Oliveros J, Del Canto-Díaz M, Alobera-Gracia MA, Del Canto-Pingarrón M, Martínez-González JM (2019). Use of autologous tooth-derived graft material in the post-extraction dental socket. Pilot study. Med Oral Patol Oral Cir Bucal.

