# Supplementary material for: The use of autogenous tooth bone graft is an efficient method of alveolar ridge preservation – meta-analysis and systematic review
Source: BMC Oral Health. 2023 Apr 19;23:226. doi: 10.1186/s12903-023-02930-2 (PMC10116659; doi:10.1186/s12903-023-02930-2)
Supplement: Supplementary file 1 — Additional file 1: Appendix Table 1. PRISMA 2020 Main Checklist. Appendix Table 2. Certainty of evidence for each meta-analysis based on the GRADE approach. Appendix Table 3. Changes in alveolar ridge width (mean ± standard deviation [SD]). Raw data of Radoczy Drajko et al. was also used (measurement at the crestal level). Appendix Table 4. Histological outcomes (mean ± standard deviation [SD]). Appendix Figure 1. Quality assessment based on Cochrane Risk of Bias Tool 2 (RoB 2) for randomized controlled trials. Appendix Figure 2. Quality assessment based on Cochrane risk of bias tool to assess non-randomized studies of interventions (ROBINS-I) for non-randomized studies of interventions. Appendix Figure 3. Funnel plots indicates the presence of statistical heterogeneity and cofounding factors affecting the primary outcome (alveolar ridge width changes). Appendix Figure 4. Funnel plots indicates the presence of statistical heterogeneity and cofounding factors affecting the residual graft proportion (%). Appendix Figure 5. Funnel plots indicates the presence of statistical heterogeneity and cofounding factors affecting the newly formed bone proportion (%). Appendix Figure 6. Funnel plots indicates the presence of statistical heterogeneity and cofounding factors affecting the connective tissue proportion (%). Appendix Figure 7. Pooled mean of residual graft proportion. Neither clinical, nor statistical significant difference can be observed between the subgroups. Appendix Figure 8. Pooled mean of newly formed bone proportion. A statistical significant difference can be observed between the subgroups. Appendix Figure 9. Pooled mean of connective tissue proportion. No statistical significant difference can be observed between the subgroups. Appendix Document 1. Search key. Appendix Document 2. Detailed statistical analysis. Appendix document 3. Detailed study charachteristics. [file 12903_2023_2930_MOESM1_ESM.docx]

**Supplementary material**

**Additional file 1: Appendix Table 1** PRISMA 2020 Main Checklist.

#

| **Topic** | **No.** | **Item** | **Location where item is reported** |
| --- | --- | --- | --- |
| **TITLE** |  |  |  |
| **Title** | 1 | Identify the report as a systematic review. | 1 |
| **ABSTRACT** |  |  |  |
| **Abstract** | 2 | See the PRISMA 2020 for Abstracts checklist |  |
| **INTRODUCTION** |  |  |  |
| **Rationale** | 3 | Describe the rationale for the review in the context of existing knowledge. | 5-6 |
| **Objectives** | 4 | Provide an explicit statement of the objective(s) or question(s) the review addresses. | 6 |
| **METHODS** |  |  |  |
| **Eligibility criteria** | 5 | Specify the inclusion and exclusion criteria for the review and how studies were grouped for the syntheses. | 7,8 |
| **Information sources** | 6 | Specify all databases, registers, websites, organisations, reference lists and other sources searched or consulted to identify studies. Specify the date when each source was last searched or consulted. | 7 |
| **Search strategy** | 7 | Present the full search strategies for all databases, registers and websites, including any filters and limits used. | 7 |
| **Selection process** | 8 | Specify the methods used to decide whether a study met the inclusion criteria of the review, including how many reviewers screened each record and each report retrieved, whether they worked independently, and if applicable, details of automation tools used in the process. | 7 |
| **Data collection process** | 9 | Specify the methods used to collect data from reports, including how many reviewers collected data from each report, whether they worked independently, any processes for obtaining or confirming data from study investigators, and if applicable, details of automation tools used in the process. | 7 |
| **Data items** | 10a | List and define all outcomes for which data were sought. Specify whether all results that were compatible with each outcome domain in each study were sought (e.g. for all measures, time points, analyses), and if not, the methods used to decide which results to collect. | 7,8 |
|  | 10b | List and define all other variables for which data were sought (e.g. participant and intervention characteristics, funding sources). Describe any assumptions made about any missing or unclear information. | 9 |
| **Study risk of bias assessment** | 11 | Specify the methods used to assess risk of bias in the included studies, including details of the tool(s) used, how many reviewers assessed each study and whether they worked independently, and if applicable, details of automation tools used in the process. | 8 |
| **Effect measures** | 12 | Specify for each outcome the effect measure(s) (e.g. risk ratio, mean difference) used in the synthesis or presentation of results. | 8 |
| **Synthesis methods** | 13a | Describe the processes used to decide which studies were eligible for each synthesis (e.g. tabulating the study intervention characteristics and comparing against the planned groups for each synthesis (item 5)). | 8 |
|  | 13b | Describe any methods required to prepare the data for presentation or synthesis, such as handling of missing summary statistics, or data conversions. | 8 |
|  | 13c | Describe any methods used to tabulate or visually display results of individual studies and syntheses. | 8 |
|  | 13d | Describe any methods used to synthesize results and provide a rationale for the choice(s). If meta-analysis was performed, describe the model(s), method(s) to identify the presence and extent of statistical heterogeneity, and software package(s) used. | 8, supplementary material |
|  | 13e | Describe any methods used to explore possible causes of heterogeneity among study results (e.g. subgroup analysis, meta-regression). | 8, supplementary material |
|  | 13f | Describe any sensitivity analyses conducted to assess robustness of the synthesized results. | 8, supplementary material |
| **Reporting bias assessment** | 14 | Describe any methods used to assess risk of bias due to missing results in a synthesis (arising from reporting biases). | 9 |
| **Certainty assessment** | 15 | Describe any methods used to assess certainty (or confidence) in the body of evidence for an outcome. | 9 |
| **RESULTS** |  |  |  |
| **Study selection** | 16a | Describe the results of the search and selection process, from the number of records identified in the search to the number of studies included in the review, ideally using a flow diagram. | 9 |
|  | 16b | Cite studies that might appear to meet the inclusion criteria, but which were excluded, and explain why they were excluded. | 12, 13 |
| **Study characteristics** | 17 | Cite each included study and present its characteristics. | 9, supplementary material |
| **Risk of bias in studies** | 18 | Present assessments of risk of bias for each included study. | figures 2, 3 |
| **Results of individual studies** | 19 | For all outcomes, present, for each study: (a) summary statistics for each group (where appropriate) and (b) an effect estimate and its precision (e.g. confidence/credible interval), ideally using structured tables or plots. | Table 1-3 |
| **Results of syntheses** | 20a | For each synthesis, briefly summarise the characteristics and risk of bias among contributing studies. | 9, Figure 2,3 |
|  | 20b | Present results of all statistical syntheses conducted. If meta-analysis was done, present for each the summary estimate and its precision (e.g. confidence/credible interval) and measures of statistical heterogeneity. If comparing groups, describe the direction of the effect. | 10-11 |
|  | 20c | Present results of all investigations of possible causes of heterogeneity among study results. | 10-11 |
|  | 20d | Present results of all sensitivity analyses conducted to assess the robustness of the synthesized results. | 10-11 |
| **Reporting biases** | 21 | Present assessments of risk of bias due to missing results (arising from reporting biases) for each synthesis assessed. | Figure 1 |
| **Certainty of evidence** | 22 | Present assessments of certainty (or confidence) in the body of evidence for each outcome assessed. | 9, Supplementary table 1 |
| **DISCUSSION** |  |  |  |
| **Discussion** | 23a | Provide a general interpretation of the results in the context of other evidence. | 12-13 |
|  | 23b | Discuss any limitations of the evidence included in the review. | 12 |
|  | 23c | Discuss any limitations of the review processes used. | 13 |
|  | 23d | Discuss implications of the results for practice, policy, and future research. | 13 |
| **OTHER INFORMATION** |  |  |  |
| **Registration and protocol** | 24a | Provide registration information for the review, including register name and registration number, or state that the review was not registered. | 7 |
|  | 24b | Indicate where the review protocol can be accessed, or state that a protocol was not prepared. | 7 |
|  | 24c | Describe and explain any amendments to information provided at registration or in the protocol. | 7 |
| **Support** | 25 | Describe sources of financial or non-financial support for the review, and the role of the funders or sponsors in the review. | 2 |
| **Competing interests** | 26 | Declare any competing interests of review authors. | 2 |
| **Availability of data, code and other materials** | 27 | Report which of the following are publicly available and where they can be found: template data collection forms; data extracted from included studies; data used for all analyses; analytic code; any other materials used in the review. | template data collection forms, data used for all analyses |

#####

# PRIMSA Abstract Checklist

| **Topic** | **No.** | **Item** | **Reported?** |
| --- | --- | --- | --- |
| **TITLE** |  |  |  |
| **Title** | 1 | Identify the report as a systematic review. | Yes |
| **BACKGROUND** |  |  |  |
| **Objectives** | 2 | Provide an explicit statement of the main objective(s) or question(s) the review addresses. | Yes |
| **METHODS** |  |  |  |
| **Eligibility criteria** | 3 | Specify the inclusion and exclusion criteria for the review. | No |
| **Information sources** | 4 | Specify the information sources (e.g. databases, registers) used to identify studies and the date when each was last searched. | Yes |
| **Risk of bias** | 5 | Specify the methods used to assess risk of bias in the included studies. | No |
| **Synthesis of results** | 6 | Specify the methods used to present and synthesize results. | Yes |
| **RESULTS** |  |  |  |
| **Included studies** | 7 | Give the total number of included studies and participants and summarise relevant characteristics of studies. | Yes |
| **Synthesis of results** | 8 | Present results for main outcomes, preferably indicating the number of included studies and participants for each. If meta-analysis was done, report the summary estimate and confidence/credible interval. If comparing groups, indicate the direction of the effect (i.e. which group is favoured). | Yes |
| **DISCUSSION** |  |  |  |
| **Limitations of evidence** | 9 | Provide a brief summary of the limitations of the evidence included in the review (e.g. study risk of bias, inconsistency and imprecision). | Yes |
| **Interpretation** | 10 | Provide a general interpretation of the results and important implications. | Yes |
| **OTHER** |  |  |  |
| **Funding** | 11 | Specify the primary source of funding for the review. | Yes |
| **Registration** | 12 | Provide the register name and registration number. | Yes |

*From:* Page MJ, McKenzie JE, Bossuyt PM, Boutron I, Hoffmann TC, Mulrow CD, et al. The PRISMA 2020 statement: an updated guideline for reporting systematic reviews. MetaArXiv. 2020, September 14. DOI: 10.31222/osf.io/v7gm2. For more information, visit: [www.prisma-statement.org](file:///C:\Users\Eleonóra\Downloads\www.prisma-statement.org)

**Additional file 1: Appendix Table 2** Certainty of evidence for each meta-analysis based on the GRADE approach.

| **Baseline compared to Autologous tooth bone graft for Alveolar ridge preservation**  **Bibliography:** | | | | | | | | | | | |
| --- | --- | --- | --- | --- | --- | --- | --- | --- | --- | --- | --- |
| **Certainty assessment** | | | | | | | **Summary of findings** | | | | |
| **Participants (studies) Follow-up** | **Risk of bias** | **Inconsistency** | **Indirectness** | **Imprecision** | **Publication bias** | **Overall certainty of evidence** | **Study event rates (%)** | | **Relative effect (95% CI)** | **Anticipated absolute effects** | |
|  |  |  |  |  |  |  | **With Autologous tooth bone graft** | **With Baseline** |  | **Risk with Autologous tooth bone graft** | **Risk difference with Baseline** |
| **Ridge width changes (mm) (follow-up: range 3 months to 6 months)** | | | | | | | | | | | |
| 103 (6 RCTs) | not serious | not serious | serious^a^ | serious^b^ | none | ⨁⨁◯◯ Low |  | 103 | - | The mean ridge width changes (mm) was **0** | mean **0.72 lower** (1.03 lower to 0.42 lower) |
| **Residual graft proportion (%) (follow-up: range 3 months to 6 months; assessed with: percentage)** | | | | | | | | | | | |
| 96 (6 RCTs) | not serious | serious^c^ | not serious | serious^b^ | strong association | ⨁⨁⨁◯ Moderate |  | 96 | - | The mean residual graft proportion (%) was **0** | mean **11.61 higher** (9.05 higher to 14.17 higher) |
| **Newly formed bone proportion (%) (follow-up: range 3 months to 6 months)** | | | | | | | | | | | |
| 96 (6 RCTs) | not serious | serious^c^ | not serious | serious^b^ | none | ⨁⨁◯◯ Low |  | 96 | - | The mean newly formed bone proportion (%) was **0** % | MD **40.23 % more** (33.04 more to 47.42 more) |
| **Connective tissue proportion (%) (follow-up: range 3 months to 6 months)** | | | | | | | | | | | |
| 96 (6 RCTs) | not serious | serious^c^ | not serious | serious^b^ | none | ⨁⨁◯◯ Low |  | 96 | - | The mean connective tissue proportion (%) was **0** | mean **45.39 higher** (38.48 higher to 52.31 higher) |

**Additional file 1: Appendix Table 3** Changes in alveolar ridge width (mean ± standard deviation [SD]). Raw data of Radoczy Drajko et al. was also used (measurement at the crestal level).

| **Measurement at the crestal level** | | |
| --- | --- | --- |
| **Author and year** | **mean difference** | **SD** |
| **I. W. Um et al. 2019** | -0.67 | 0.89 |
| **C. P. Joshi et al.  2016** | -0.15 | 0.08 |
| **A. Dwivedi et al.,  2020** | -0.68 | 1.05 |
| **Z. Radoczy-Drajko et al.  2021** | -0.69 | 1.64 |
| **effect size** | **- 0.46** | **CI [-0.81; -0.10]** |
|  |  |  |
| **Measurement 1 mm apical to the crestal level** | | |
| **Z. Radoczy-Drajko et al.  2021** | -1.68 | 1.60 |
| **A. Elfana et al.,  2021** | -1.02 | 0.45 |
| **A. Elfana et al.,  2021** | -0.85 | 0.45 |
| **G. U. Jung et al., 2018** | -0.78 | 1.69 |
| **effect size** | **-0.95 mm** | **CI [-1.15; -0.76]** |
| **overall effect size** | **-0.72 mm** | **CI [-1.03; -0.42]** |

**Additional file 1: Appendix Table 4** Histological outcomes (mean ± standard deviation [SD]).

|  | **Author and year** | **Residual graft (%)** | | **Newly formed bone (%)** | | **Connective tissue (%)** | |
| --- | --- | --- | --- | --- | --- | --- | --- |
|  |  | **mean** | **SD** | **mean** | **SD** | **mean** | **SD** |
| **Demineralized** |  |  |  |  |  |  |  |
|  | **I. W. Um et al. 2019** | 16.15 | 17.70 | 29.75 | 12.62 | 35.87 | 17.10 |
|  | **G. U. Jung et al., 2018** | 10.72 | 9.83 | 32.88 | 14.48 | 56.40 | 8.58 |
|  | **K. M. Pang et al., 2017** | 8.95 | 17.70 | 31.24 | 13.87 | 59.81 | 15.50 |
| **effect size** |  | 9.55 % | 95% CI  [ 7.19; 11.91] | 31.17% | 95% CI  [ 26.99; 35.35] | 51.29% | 95% CI  [ 37.29; 65.29] |
|  |  |  |  |  |  |  |  |
| **Partially-demineralized** | **Z. Radoczy-Drajko et al.  2021** | 7.44 | 6.64 | 55.67 | 15.11 | 36.89 | 13.96 |
|  | **A. Elfana et al.,  2021** | 11.45 | 4.13 | 48.40 | 11.56 | 40.15 | 7.73 |
| **effect size** |  | 9.84% | 95% CI  [ 5.99; 13.69] | 51.21% | 95% CI  [ 44.27; 58.15] | 39.44% | 95% CI  [ 35.20; 43.69] |
|  |  |  |  |  |  |  |  |
| **Un--demineralized** | **A. Santos et al.,  2021** | 12.20 | 7.70 | 47.30 | 14.80 | 40.50 | 17.60 |
|  | **A. Elfana et al.,  2021** | 17.05 | 5.58 | 37.50 | 8.94 | 45.40 | 4.06 |
| **effect size** |  | 14.54% | 95% CI [ 9.79; 19.29 | 42.38% | 95% CI  [ 32.77; 51.98] | 42.38% | 95% CI  [ 39.66; 48.34] |
| **overall effect size** |  | **11.61%** | **95% CI  [ 9.05; 14.17]** | **40.23%** | **95% CI  [ 33.04; 47.42]** | **45.39%** | **95% CI  [ 38.48; 52.31]** |

**Additional file 1: Appendix Figure 1** Quality assessment based on Cochrane Risk of Bias Tool 2 (RoB 2) for randomized controlled trials.

**Additional file 1: Appendix Figure 2** Quality assessment based on Cochrane risk of bias tool to assess non-randomized studies of interventions (ROBINS-I) for non-randomized studies of interventions.

**Additional file 1: Appendix Figure 3** Funnel plots indicates the presence of statistical heterogeneity and cofounding factors affecting the primary outcome (alveolar ridge width changes).

**
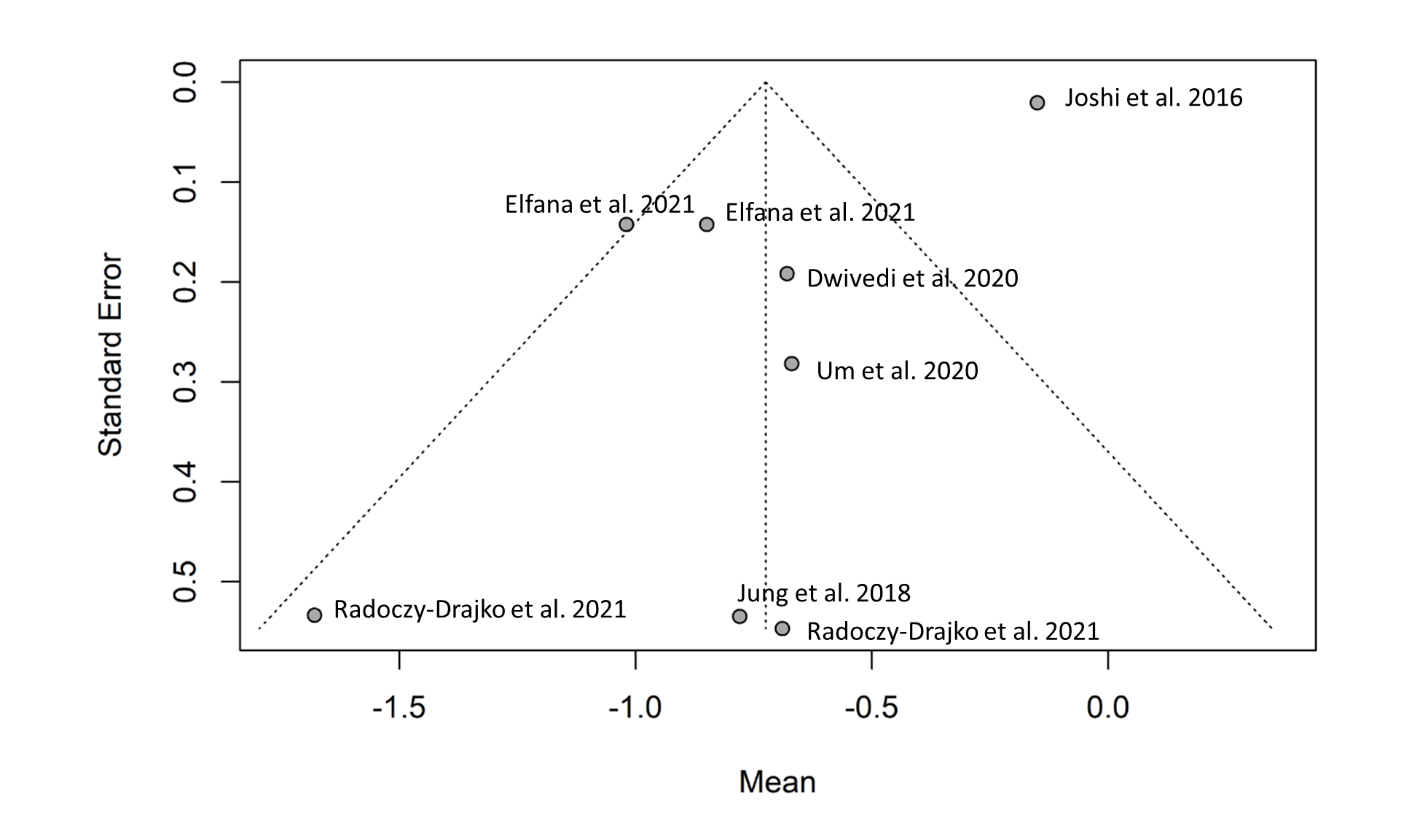
**

**Additional file 1: Appendix Figure 4** Funnel plots indicates the presence of statistical heterogeneity and cofounding factors affecting the residual graft proportion (%).

**
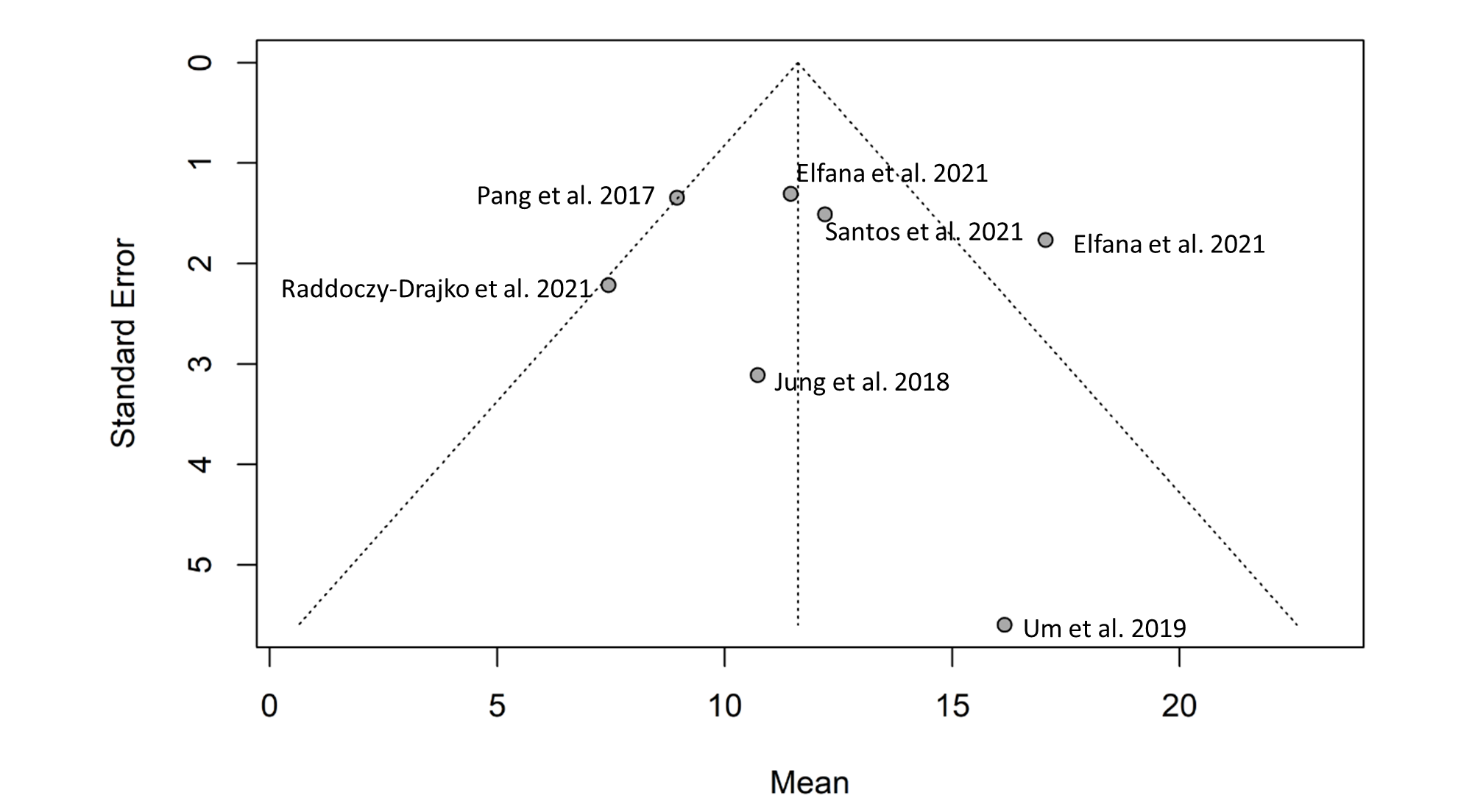
**

**Additional file 1: Appendix Figure 5** Funnel plots indicates the presence of statistical heterogeneity and cofounding factors affecting the newly formed bone proportion (%).

**
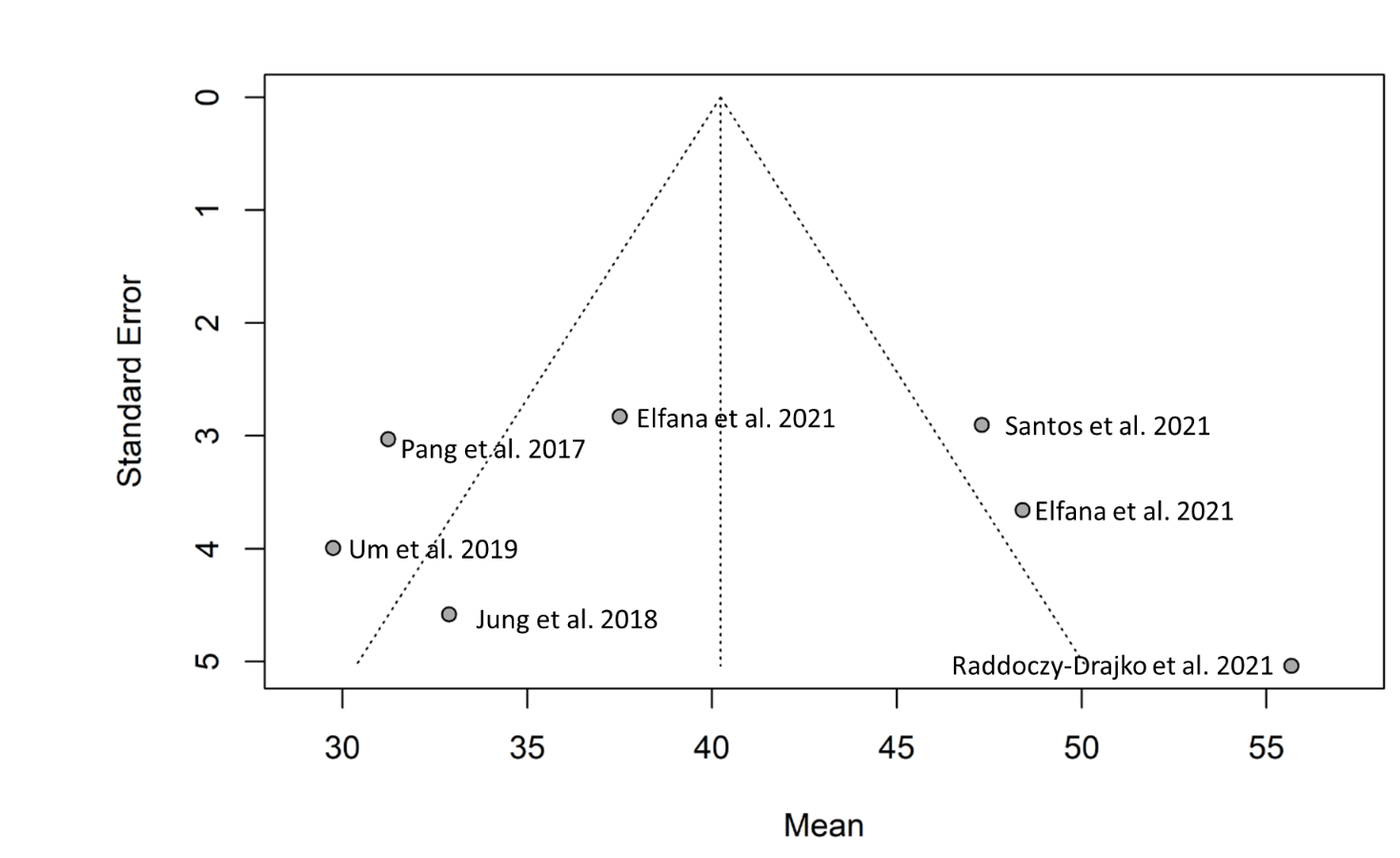
**

**Additional file 1: Appendix Figure 6** Funnel plots indicates the presence of statistical heterogeneity and cofounding factors affecting the connective tissue proportion (%).

**
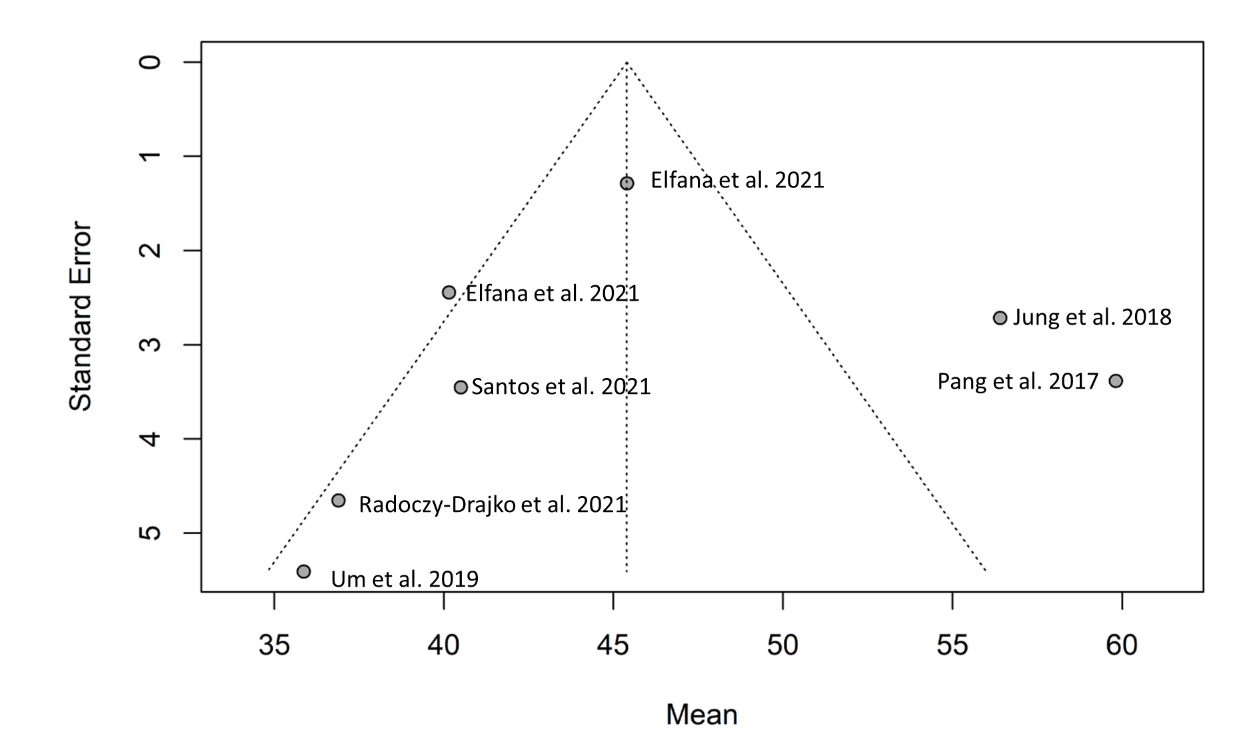
**

**Additional file 1: Appendix Figure 7** Pooled mean of residual graft proportion. Neither clinical, nor statistical significant difference can be observed between the subgroups.

**
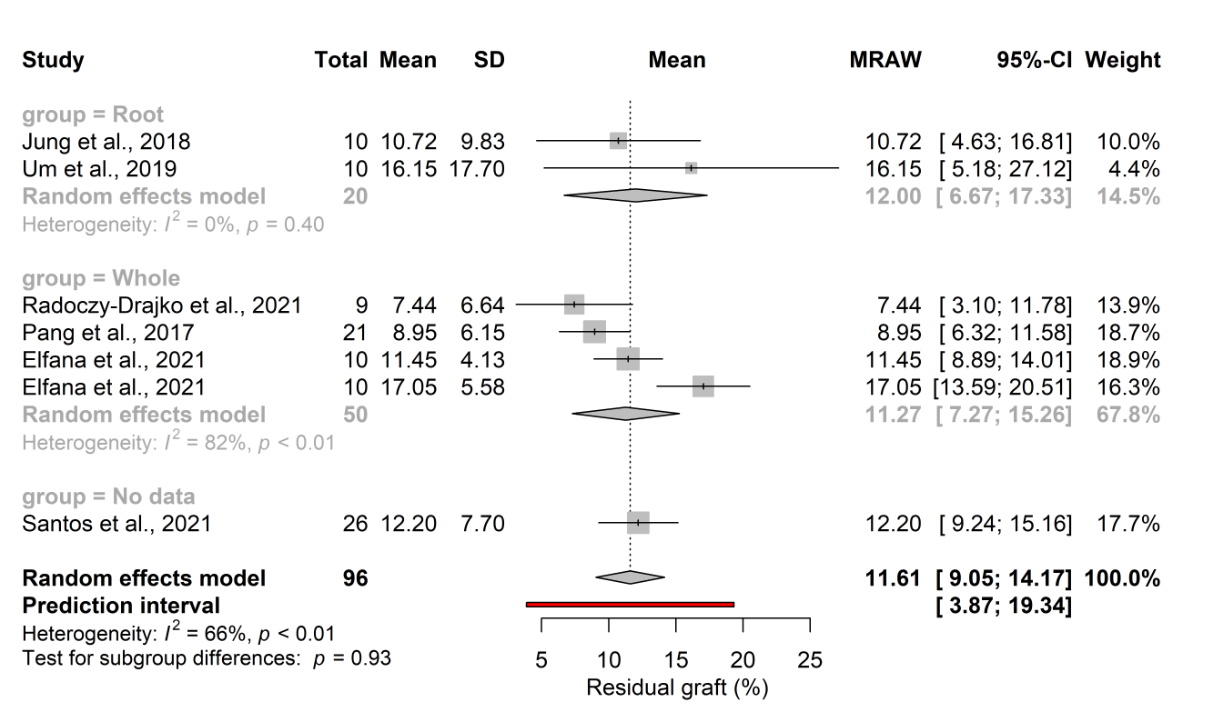
**

**Additional file 1: Appendix Figure 8** Pooled mean of newly formed bone proportion. A statistical significant difference can be observed between the subgroups.


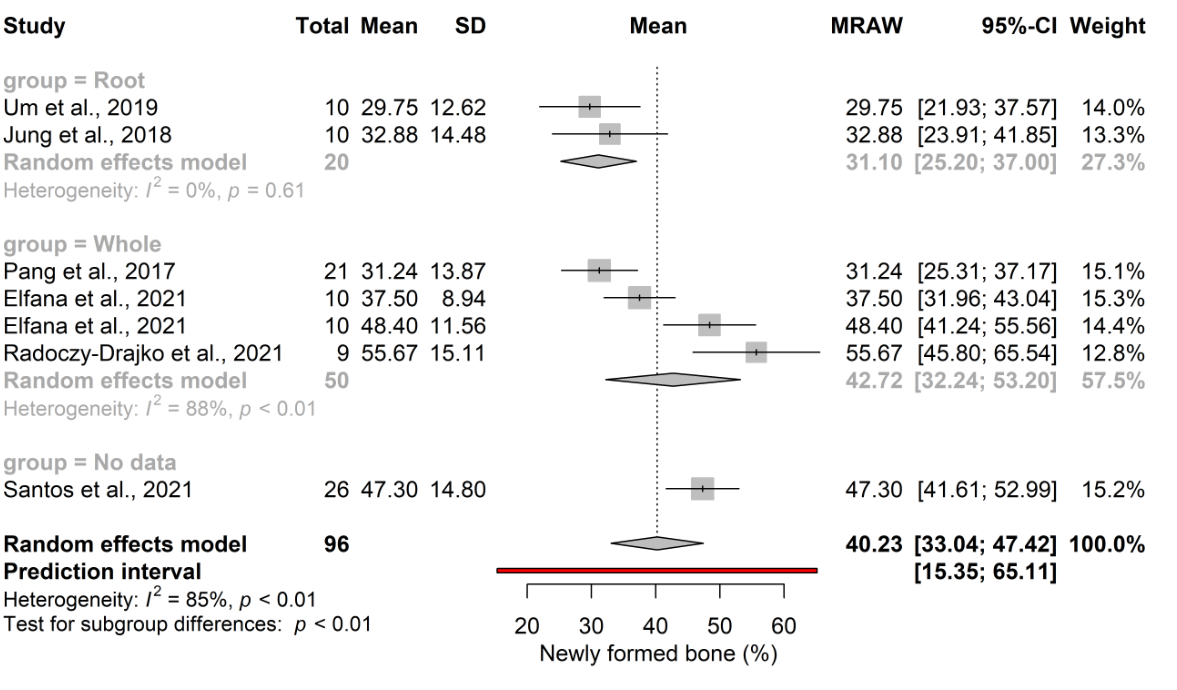


**Additional file 1: Appendix Figure 9** Pooled mean of connective tissue proportion. No statistical significant difference can be observed between the subgroups.

**
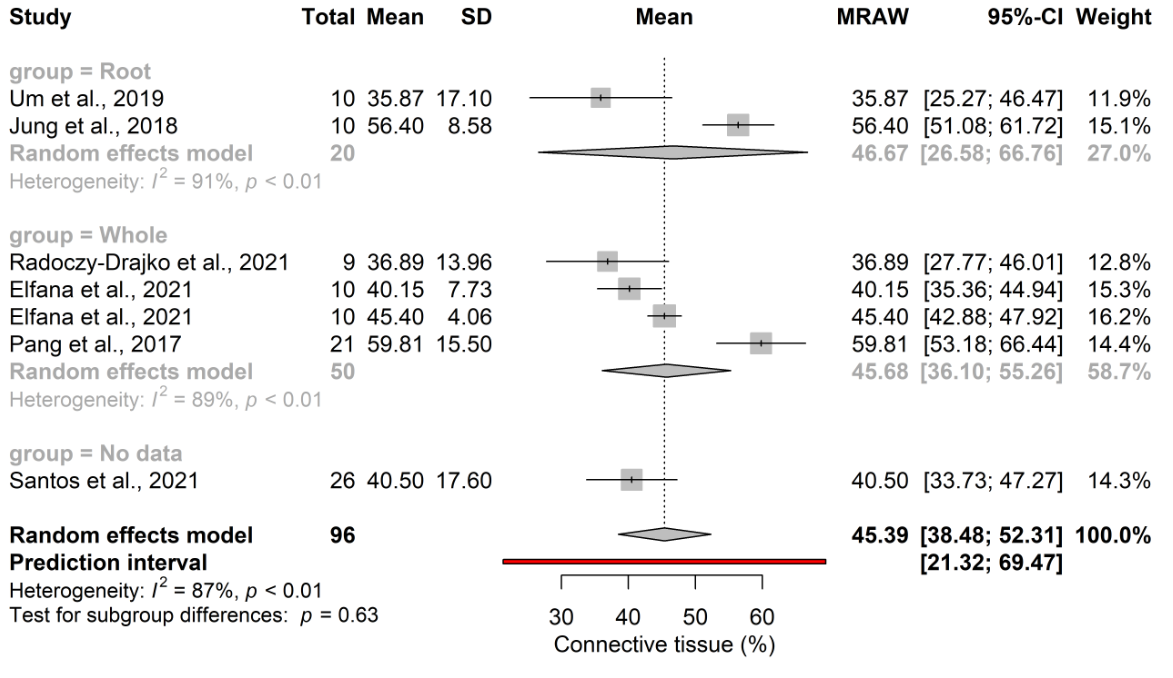
**

**Additional file 1: Appendix Document 1. Search key**

("Socket preservation" OR "alveolar ridge preservation" OR ARP OR "alveolar preservation" OR "socket grafting" OR extract* tooth OR extract* teeth) AND (autogen* graft OR autolog* graft) AND ("human dentin" OR "dentin matrix" OR "dentin graft" OR ATB OR AutoBT OR ATG OR "tooth graft" OR teeth graft OR teeth derived graft OR tooth derived graft OR autologous tooth bone graft OR "Autologous Tooth Structure" OR autogen* tooth bone graft).

**Additional file 1: Appendix Document 2. Detailed statistical analysis**

Both primary and secondary outcomes are continuous data. The change in ridge width represents horizontal reduction of the crest at the top of the ridge, as well as 1mm apically, relative to the initial values measured in millimetres, while the percentage of different components in histological samples ranges from 0 to 100. So far, only a few studies investigated ATB as a grafting material, and in these analyses different comparators were used, so we could not collect a common control group for analysis.

Due to the considerable between-study heterogeneity, we used a random effects model to pool the effect sizes. Inverse variance weighting method was used to calculate the pooled means. For outcomes where the study number was more than five, a Hartung-Knapp adjustment (IntHout et al., 2014; Knapp & Hartung, 2003) was applied. No adjustment was applied for fewer than five studies. Between-study heterogeneity was described by means of Cochrane Q test, and the Higgins&Thompson’s *I*^2^ statistics (Thompson & Higgins, 2002).

Forest plots were used to graphically summarize the results. The confidence intervals of the individual studies and the pooled results were calculated based on the t-distribution. Where applicable, we reported the prediction intervals (i.e. the expected range of effects of future studies) of results following the recommendations of IntHout et al. Due to the low number of studies, precise outlier and influence analyses were not possible to be carried out. Subgroups were defined based on the different graft properties (demineralized / partially demineralized / undemineralized). In case of subgroup analysis a mixed-effects model were used. All subgroups were assumed to share a common *τ*^2^ value as the difference was not anticipated in the between-study heterogeneity in the subgroups and the study number was relatively small in some subgroups. To assess the difference between the subgroups a Cochrane Q test was used.

**Additional file 1: Appendix document 3. Detailed study charachteristics**

**Population epidemiology, tooth types and reason for extraction**

The selected studies included a number of subjects ranging between six and 30. Two studies did not report on the average/ mean age of patients (Dwivedi & Kour, 2020; Radoczy-Drajko et al., 2021)and only one did not provide information on the gender of the participants (Radoczy-Drajko et al., 2021). Local and systematic criteria for inclusion / exclusion were clearly reported in all the papers included in the meta-analysis, however the reasons for the extraction are reported in only two studies(Jung et al., 2018; Um et al., 2019). Most studies evaluated effects of ARP on both mandibular and maxillary sites(Dwivedi & Kour, 2020; Elfana et al., 2021; Joshi et al., 2016; Jung et al., 2018; Pang et al., 2017; Radoczy-Drajko et al., 2021; Um et al., 2019). Three studies included all types of teeth(Dwivedi & Kour, 2020; Pang et al., 2017; Um et al., 2019), while Jung et al. included premolar and molar teeth, Elfana et al. included non-molar teeth (Elfana et al., 2021), and Radoczy et al. included only single rooted teeth(Radoczy-Drajko et al., 2021). Two studies do not report any information regarding tooth type (Joshi et al., 2016; Santos et al., 2021).

**Evaluation parameters and methods**

For meta-analysis, only data regarding alveolar ridge width and histomorphometric outcomes were extracted from the selected studies. In three studies, the primary outcome variable was defined as vertical and horizontal ridge alterations after the healing period (Elfana et al., 2021; Joshi et al., 2016; Um et al., 2019), while in one study the primary outcome variable was implant stability after placement (Santos et al., 2021). The other articles did not specify a primary outcome variable among the recorded parameters.
